# Adverse drug events resulting from use of drugs with sulphonamide-containing anti-malarials and artemisinin-based ingredients: findings on incidence and household costs from three districts with routine demographic surveillance systems in rural Tanzania

**DOI:** 10.1186/1475-2875-12-236

**Published:** 2013-07-11

**Authors:** Joseph D Njau, Abdulnoor M Kabanywanyi, Catherine A Goodman, John R MacArthur, Bryan K Kapella, John E Gimnig, Elizeus Kahigwa, Peter B Bloland, Salim M Abdulla, S Patrick Kachur

**Affiliations:** 1 Department of Health Policy and Management, Rollins School of Public Health (Emory University), Atlanta GA 30322, USA; 2 Ifakara Health Institute (IHI), Dar es Salaam, Tanzania; 3 London School of Hygiene and Tropical Medicine (LSHTM), London, UK; 4 Division of Parasitic Diseases and Malaria, Center for Global Health (CGH), Centers for Disease Control and Prevention (CDC), Atlanta GA 30333, USA; 5 Swiss Development Cooperation (SDC), Dar es Salaam, Tanzania

**Keywords:** Anti-malarial, Adverse drug reactions, Household costs, ACT, Sulphonamide, Tanzania

## Abstract

**Background:**

Anti-malarial regimens containing sulphonamide or artemisinin ingredients are widely used in malaria-endemic countries. However, evidence of the incidence of adverse drug reactions (ADR) to these drugs is limited, especially in Africa, and there is a complete absence of information on the economic burden such ADR place on patients. This study aimed to document ADR incidence and associated household costs in three high malaria transmission districts in rural Tanzania covered by demographic surveillance systems.

**Methods:**

Active and passive surveillance methods were used to identify ADR from sulphadoxine-pyrimethamine (SP) and artemisinin (AS) use. ADR were identified by trained clinicians at health facilities (passive surveillance) and through cross-sectional household surveys (active surveillance). Potential cases were followed up at home, where a complete history and physical examination was undertaken, and household cost data collected. Patients were classified as having ‘possible’ or ‘probable’ ADR by a physician.

**Results:**

A total of 95 suspected ADR were identified during a two-year period, of which 79 were traced, and 67 reported use of SP and/or AS prior to ADR onset. Thirty-four cases were classified as ‘probable’ and 33 as ‘possible’ ADRs. Most (53) cases were associated with SP monotherapy, 13 with the AS/SP combination (available in one of the two areas only), and one with AS monotherapy. Annual ADR incidence per 100,000 exposures was estimated based on ‘probable’ ADR only at 5.6 for AS/SP in combination, and 25.0 and 11.6 for SP monotherapy. Median ADR treatment costs per episode ranged from US$2.23 for those making a single provider visit to US$146.93 for patients with four visits. Seventy-three per cent of patients used out-of-pocket funds or sold part of their farm harvests to pay for treatment, and 19% borrowed money.

**Conclusion:**

Both passive and active surveillance methods proved feasible methods for anti-malarial ADR surveillance, with active surveillance being an important complement to facility-based surveillance, given the widespread practice of self-medication. Household costs associated with ADR treatment were high and potentially catastrophic. Efforts should be made to both improve pharmacovigilance across Africa and to identify strategies to reduce the economic burden endured by households suffering from ADR.

## Background

The development of malaria parasite resistance to commonly used drugs such as chloroquine and sulphadoxine-pyrimethamine (SP) has complicated global efforts to mitigate the burden of malaria disease especially in the poorest countries [1,2]. The last decade has witnessed major changes in malaria treatment protocols with most malaria-endemic countries switching from cheap and ineffective drugs to relatively expensive but more efficacious artemisinin-based combination therapy (ACT) [3,4]. ACT has been hailed by the World Health Organization (WHO) as the best new hope for malaria treatment in Africa and other endemic regions [5]. Until the end of the 1990s, experience with large-scale use of ACT was mainly confined in Southeast Asia [6]. At the turn of the new century, larger scale ACT trials began in other parts of the world including sub-Saharan Africa [7,8]. By 2011, 79 countries and territories in malaria-endemic regions had adopted ACT as first-line treatment for *Plasmodium falciparum* malaria [9]. Meanwhile, SP continues to be used as a drug of choice for intermittent presumptive treatment for malaria in pregnancy (IPTp). Nevertheless, sulphonamide medicines are widely used as antibiotic regimens for acute respiratory infections, urinary tract infections and as prophylaxis for HIV + patients [10,11]. The use of sulphonamide-containing anti-malarials and other anti-malarial drugs with artemisinin ingredients has in the last decade become widespread [12].

Adverse drug reactions (ADR) can be defined as any drug action that is not of diagnostic, therapeutic or prophylactic benefit to the user [13]. To date, most studies documenting the incidence of ADR caused by sulphonamides have been outside Africa [14-17]. Sulphonamide-containing anti-malarials have been found to cause severe adverse reactions, including severe skin rash (toxic epidermal necrolysis (TEN) and Stevens-Johnson syndrome (SJS)), liver disorders, and bone marrow suppression [15]. Furthermore, the spread of HIV infection is thought to have increased the number of patients experiencing sulpha-related reactions because of multiple drug use in the context of a compromised immune system [18,19]. Conflicting results associating overdose of artemisinin containing drugs with unusual and selective patterns of damage to certain brainstem nuclei in animals and humans have also been reported [20-23].

Concerns about the incidence of ADR in sub-Saharan Africa have increased for a number of reasons. First, there is widespread self-medication and presumptive treatment of malaria, which tends to lead to over- prescription of anti-malarials for febrile illnesses. Second, there are concerns about proliferation of substandard and counterfeit medicines, exacerbated by weak national regulatory authorities [24-26]. The third reason relates to increasing numbers of new anti-malarials entering the market in the context of an almost complete lack of phase IV post-marketing surveillance in the region [27,28].

Because of these concerns, public health researchers and policy makers are supporting efforts to establish effective and reliable drug surveillance programmes in Africa [27-29]. However, only a few studies have investigated the frequency of anti-malarial ADR in sub Saharan Africa [30-32]. This study aims to address this gap. It is a component of the Interdisciplinary Monitoring Programme for Anti-malarial Combination Therapy (IMPACT-Tz) which was jointly designed and implemented by the Ifakara Health Institute (IHI) and the US Centers for Disease Control and Prevention (CDC) in collaboration with the Government of Tanzania [33]. The study implemented both passive and active ADR surveillance systems in health and demographic surveillance sites (HDSS) covering parts of the three districts of Rufiji, Kilombero and Ulanga [34]. The HDSS sites cover a combined population of over 157,000, roughly about 22% of the total population residing in the three districts (estimated at 716,892 in 2002) [35]. Three times a year, HDSS staff visit households sampled from a cluster of 56 enumerated villages to collect data on health, demographic and socio-economic indicators.

The aim of the ADR surveillance system was to document both household-level incidence and economic burden of ADR resulting from use of anti-malarials. The study used community and health facility-based surveillance to register and follow up all potential ADR resulting from use of sulphonamide- and artemisinin-containing anti-malarials. This investigation covered over 157,000 individuals and successfully completed 24 months of clinical surveillance on ADR events caused by use of SP and/or AS anti-malarials in moderate to high transmission settings in rural Tanzania. Additionally the study provides the first data on the cost burden incurred by households in treatment of patients with anti-malarial ADR. This information has two key uses: first, it should help prioritize pharmacovigilance activities and the provision of services to ADR sufferers; and secondly, these cost estimates can contribute to large cost-effectiveness analyses of, for example, changing national drug policy as well as providing more accurate and complete estimates of the household economic burden of malaria.

## Methods

### Description of the study sites

This study was part of a multi-year project involving evaluation of large-scale use of anti-malarial combination drugs implemented in three rural districts of Kilombero, Ulanga, and Rufiji located in Morogoro and Coastal Regions in south-eastern Tanzania. The project is described in detail elsewhere [36,37]. The districts are geographically contiguous, but the Rufiji population is separated from those of Kilombero and Ulanga by the Selous Game Reserve. The most common occupation in the study areas is subsistence farming, with rice, maize and cassava being predominant food crops. Other economic activities include animal husbandry, fishing and small-scale trading. The districts have very limited paved roads and some villages are cut off from major market centres during rainy seasons. At the time this study was being conducted, malaria transmission was year round intense with very high estimated mean entomological inoculation rate of between 79 and 1,209 infective bites per person per year [38]. Malaria remains the leading cause of outpatient diagnoses, and reported inpatient deaths.

Health care is provided by a network of public and faith-based health facilities, including hospitals, health centres and dispensaries, but a significant portion of care is also provided by private sources, predominately drug shops and kiosks [39]. Moreover, studies have shown that up to 40% of patients seek care from private sources [37,40].

In Tanzania, anti-malarial treatment policy was changed in August 2001 when chloroquine was replaced with SP as first-line drug for uncomplicated *P*. *falciparum* malaria. By this time chloroquine treatment failures had reached a record level of over 60% of all outpatient malaria cases [41]. The switch to SP led to widespread public concern due to fear of ADR, which was extensively covered in the media, leading many people to avoid SP use [42]. From 2003 through 2006 the IMPACT-Tz project piloted district-wide ACT use in Rufiji, using the combination of SP + artesunate (SP/AS). As evidence of increasing SP clinical failures accumulated, the national first-line policy was changed again after only five years, with the ACT artemether-lumefantrine (ALu) being nationally adopted as first-line drug in 2006 [43]. To accomplish this study, data collection was carried out during the year 2003 to 2005. Therefore, during the period of data collection for this study, the first-line anti-malarial was SP monotherapy in Kilombero and Ulanga, and AS/SP in Rufiji. For this reason, the ADR surveillance findings are presented separately for Rufiji District. During the same period, approximately 58% of patients with febrile illnesses were given anti-malarials upon their visit to health facilities or drug outlets.

### Data collection

ADR cases were identified through both *passive* and *active* surveillance. For the passive surveillance system from February 2003 clinical personnel in public and faith-based health facilities in the HDSS were trained on the signs and causes of ADR reactions. At least one health worker from a network of one hospital, four health centres and 28 dispensaries was trained to recognize, treat, document and report ADR cases presenting at their health facilities. The majority of trained health practitioners were clinical officers and nurses, with a few general physicians. Colour photographs of patients with SJS/TEN were provided and potential ADR were discussed in detail.

A screening case definition for suspected severe cutaneous ADR was defined as having at least two of the following symptoms: (i) a widespread, disseminated rash, (ii) blisters or skin detachment greater than 3 cm in diameter, or (iii) lesions on at least one mucous membrane. Additionally, clinicians were instructed to report all patients suffering from ataxia, impaired fine finger dexterity, balance problems, facial oedema, shortness of breath, urticarial rashes, or if the patient felt lightheaded within 30 min of taking the medication. Moreover, following active surveillance implementation (see below), additional symptoms of vomiting, stomach ache, diarrhoea and headache were added to the standard protocol definition after these were reported on multiple case follow-up visits.

Clinicians were asked to document clinical and demographic information of patients presenting with ADR symptoms on standard Tanzania Food and Drug Authority (TFDA) adverse event reporting forms and in specially provided log books. These reports were forwarded to the District Health Management Teams (DHMTs) and the Project ADR Surveillance Officer (ADR SO) for follow-up investigation. All ADR SOs were medical professionals with background in clinical training as clinical officers and/or assistant medical officers. Standard screening procedure based on review of ADR clinical literature and the expert advice from SJS dermatologist was developed and adopted for training the ADR SOs to assess and categorize ADR events. Additionally, a set of standard questions based on many years of field data collection experience in this area, were jointly developed by IHI and CDC Malaria Branch researchers to help clinicians ascertain any additional side-effects not captured in the standard protocol. The ADR SO conducted a complete history and physical examination of each patient with particular attention on the drug-use history and dermatologic examination. In the event of fatalities, the parent or caretaker of the patient was interviewed. This study reports on ADR events detected through passive surveillance, from January 2004 to December 2005.

To increase the number of cases reported, passive surveillance was supplemented by active case detection in 2004 and 2005. Questions on uptake of anti-malarials, particularly SP and/or AS, in the past 90 days and any related side effects were asked through cross-sectional household surveys completed in 2004 and 2005. The surveys were conducted from June to September of each year, capturing the typical high malaria transmission season in the study area. For each year’s survey, independent samples from the same clusters of 56 census enumerated villages (31 in Rufiji and 25 in Kilombero/Ulanga) were randomly selected [37]. Following informed consent, questionnaires were administered to a total of 16,290 individuals from over 5,300 households in the two years combined. Of those interviewed, 46.5% were from Rufiji and the remaining 53.5% were from Kilombero/Ulanga. Respondents were asked whether they, or their children, experienced specified side effects defined in the study protocol following their SP and/or AS intake during the last three months. Respondents were also asked to list other side effects/experiences related to these drugs. The ADR SO reviewed the completed questionnaires and selected all potential ADR cases for detailed clinical examination. Each suspected ADR case reported through active surveillance was visited at home by the ADR SO after the completion of each round of the household survey. As with cases identified through passive surveillance, the ADR SO conducted a detailed history and physical examination of ADR patients and interviewed the patient or their caretaker/close relatives. To avoid double counting by active and passive surveillance systems, the ADR SOs contacted clinicians at local health facilities to harmonize their information.

All completed case follow-up reports were forwarded to study physicians who classified the suspected cases into one of five categories: i) probable or ii) possible severe adverse reaction (TEN/SJS and/or severe neurological reaction and/or anaphylaxis) associated with sulphonamide and/or artesunate intake; iii) probable or iv) possible severe adverse reaction (TEN/SJS and/or severe neurological reaction and/or anaphylaxis) not associated with sulphonamide and/or artesunate intake; or (v) skin condition, neurological reaction and/or anaphylactic reaction not consistent with an expected side effect from sulphonamide-containing anti-malarial and/or artesunate drug intake. Side effects like headaches, diarrhoea and nausea that did not occur concurrently with the ADR symptoms (i.e. skin conditions including lesions and blisters) were not included in the ADR incidence calculations but their related household treatment costs were accounted because of their economic significance. A patient was considered exposed if s/he reported any use of SP and/or AS in the 30 days before initial symptoms of drug reactions were observed.

The interviews for all suspected ADR cases identified included questions on care-seeking and household treatment costs. Household cost data collected covered both health facility based expenditures (drugs, registration/consultation, inpatient admission, laboratory test expenses, informal payments), and non-facility expenditures (travel costs, food and accommodation costs for patients and those accompanying them to providers). Respondents incurring cash expenditures were asked how these expenses were paid for. The study did not attempt to estimate the indirect productivity costs in terms of working days lost for patients and caretakers.

### Estimation of ADR occurrence rates

To obtain the numerators for calculation of ADR occurrence rates due to exposure to SP and/or AS, events meeting the physicians’ classification of ‘probable’ ADR cases were included (‘possible’ ADR were not included in incidence estimates). For the denominator, the proportions of the population exposed to sulphonamide and artemisinin-based anti-malarial drugs was obtained through the household surveys in 2004 and 2005 described above, together with similar surveys conducted in 2001 and 2002, to help capture the variations of malaria prevalence in the study area. In these cross-sectional surveys, individuals within households were asked about their use of sulphonamide-containing anti-malarials in the past two weeks. The two weeks reported anti-malarial use was multiplied by 26 to ascertain their annual usage rates. Annualized SP and/or AS exposure rates were extrapolated for the entire HDSS study sites populations expressed as proportion of ADR occurrence per 100,000 exposures.

### Data management and analysis

Data were double entered in FOXPRO 2.6a (Microsoft Inc, Redmond, WA, USA), and analysis was performed using STATA version 10 (Stata Corp, College Station, TX, USA). Costs are presented in 2005 US$ (exchange rate US$1 = TZS1,150/=)[44].

### Ethical clearance

This study received ethical approval from the institutional review boards of the Ifakara Health Institute, the Tanzanian Medical Research Coordinating Committee and the US Centers for Disease Control and Prevention.

## Results

### Suspected ADR cases recorded through active and passive surveillance

A total of 95 suspected ADR cases were identified during the two year period (Table 1). Of these, 44 cases were recorded through passive surveillance and 51 through active surveillance. Of the reported cases, 71 (74.7%) were patients aged over five years, and 58 (60%) were women. Fifty (52.6%) of these events were recorded in Kilombero/Ulanga. A total of 79 (83.2%) cases were successfully traced for further clinical examination and ADR classification. Sixteen of the 95 cases, nine in Kilombero/Ulanga and seven in Rufiji could not be traced because of relocation, poor road accessibility and/or incorrect recording of patients’ residential information. After reviewing all traced case reports from the ADR SO, the project physician classified the events as follows: 67 (84.8%) were related to intake of SP and/or AS; eight (10.1%) cases were excluded because they occurred outside the HDSS area or were recorded before January 2004; and four (5.0%) were inconsistent with SP and/or AS intake.

**Table 1:** Adverse drug reactions reported through active and passive surveillance and dominant symptoms reported

	**Rufiji District**	**Kilombero/Ulanga Districts**^**1**^	**Total (%)**
**Total suspected adverse drug events reported**	**45**	**50**	**95 (100)**
Identified through active surveillance	21	30	51 (53.7)
Identified through passive surveillance	24	20	44 (46.3)
Number of cases successfully traced	38	41	79 (83.2)
***Of the successfully traced cases:***	**38**	**41**	**N = 79 (100)**
Events associated with SP monotherapy	20	33	53 (67.1)
Events associated with AS/SP combination therapy	13	0	13 (16.5)
Events associated with AS monotherapy	1	0	1 (1.3)
Events outside DSS area (excluded)	3	5	8 (10.0)
Events unrelated to SP and/or AS (excluded)	1	3	4 (5.0)
***Dominant symptoms reported for those included:***	**34**	**33**	**N = 67 (100)**
Body blisters and skin detachment	7	11	**18** (26.9)
Body swelling, itching and urticarial rashes	10	6	**16** (23.9)
Multiple lésions on mucus membranes	6	7	**13** (19.4)
Facial edema, sore mouth/nose conjunctiva	5	4	**9** (13.4)
Others^1^	6	5	**11** (16.4)
***Deaths***			
Deaths attributable to SP and/or AS caused ADR	2	4	**6 (8.9%)**

^1.^ These include side effects such as headache, lightheadedness, shortness of breath, stomachache, diarrhoea, vomiting and kidney pain.

The physician further classified the 67 cases associated with SP and/or AS as ‘probable’ or ‘possible’ ADR cases. A total of 34 (51%) cases were certified as ‘probable’ events and the remaining 33 (49%) as ‘possible’ (Table 2). The majority of “probable” cases (n = 29) were associated with SP monotherapy intake, with only five cases associated with the AS/SP combination, and none with AS monotherapy. Fourteen of the probable events were recorded in Rufiji where AS/SP combination drugs were being piloted, and the remaining 20 were recorded in Kilombero/Ulanga where AS was not provided. For the 33 cases classified as ‘possible’ ADR events, 24 were associated with use of SP monotherapy, eight with AS/SP intake, and only one with AS monotherapy.

**Table 2:** Classification of adverse drug reactions by type of surveillance, site and type of drugs used

	**Probable**	**Possible**	**TOTAL**
***Type of Surveillance***			
Passive surveillance	25	18	43
Active surveillance	9	15	24
***Study District***			
Rufiji	14	20	34
Kilombero/Ulanga	20	13	33
***Type of Drug Used***			
SP monotherapy	29	24	53
AS monotherapy	0	1	1
AS/SP combination	5	8	13
**TOTAL**	**34 (51%)**	**33 (49%)**	**67 (100%)**

About 78% of patients or caretakers interviewed reported experiencing/seeing multiple ADR symptoms within 36 hours of SP and/or AS intake. The dominant illness conditions reported by the 67 patients included a combination of one or multiple illness diagnostic conditions such as; body blisters and skin detachment, body swelling, itching and urticarial rashes, multiple lesions on mucous membranes around the mouths, nose or conjunctivae, and facial oedema, and other side effects like light-headedness, headache, shortness of breath, stomach ache, diarrhoea, vomiting, and also severe kidney pain (Table 1).

Four patients died at health facilities following clinical deterioration within three weeks following onset of drug reactions associated with SP use. A further three patients died outside health facilities with reported treatment of drug reactions prior to their deaths. Four of the deceased had reported SP intake, two had used the AS/SP combination and one had used sulphonamide-containing antibiotics to treat other illnesses. Two of the victims were reported to have had swollen bodies and blisters, including mouth, nose and ear membrane lesions. Other fatalities suffered multiple complications including kidney failures (n = 3), with one case exhibiting pneumonia-like symptoms. All reported deaths were classified as ‘probable’ ADR events.

### Estimation of ADR occurrence rate

ADR rate of occurrence was estimated from the 34 cases classified as ‘probable’ ADR events due to exposure to SP and/or AS (Table 3). Following the nine probable ADR events resulting from use of SP monotherapy in Rufiji HDSS, annual ADR occurrence rate per 100,000 exposures per year was estimated at 11.6 events for the Rufiji HDSS population. Annual ADR occurrence rate of 5.6 cases per every 100,000 exposures to AS/SP combination drugs used was estimated for the same population and there were no ADR cases due to AS monotherapy in Rufiji. In Kilombero and Ulanga HDSS, SP monotherapy was estimated to cause 25 ADR cases per 100,000 exposures per year (no use of AS or AS/SP combination therapy was reported in Kilombero/Ulanga).

**Table 3:** Parameters for estimation of ADR incidence from both active and passive surveillance, January 2004 to December 2005

	**RUFIJI District**	
		**KILOMBERO/ ULANGA Districts**
**Probable ADR events detected by passive surveillance:**		
SP monotherapy	7	13
Artesunate monotherapy	0	0
Combination of AS/SP	3	0
Total	10	13
**Probable ADR events detected by active surveillance:**		
SP monotherapy	2	7
Artesunate monotherapy	0	0
Combination of AS/SP	2	0
Total	4	7
**Total probable ADR events detected:**		
SP monotherapy	9	20
Artesunate monotherapy	0	0
Combination of AS/SP	5	0
Total	14	20
**Total Population Under DSS Surveillance**	84,500	74,200
**Estimates of average annual anti-malarial drug exposure rates in the DSS areas per capita:**		
SP monotherapy	0.46	0.54
Artesnuate monotherapy	0.37	0
Combination of AS/SP	0.53	0
**Estimate of total doses used by the DSS population in 2004/5** (population * average annual exposure)		
SP monotherapy	38,870	40,068
AS monotherapy	31,265	0
Combination of AS/SP	44,785	0
**Estimated ADR incidence per 100,000 exposures**^**1**^		
SP monotherapy	11.6	25.0
AS monotherapy	0	-
Combination of AS/SP	5.6	-

^1^Calculated as (Number of “probable” ADR cases / estimated total doses used) * 0.5 (multiplied by 0.5 to annualize ADR occurrence rates since our data cover a two year period).

### Care-seeking patterns

Complete costing information was available for 50 (74.6%) of the 67 cases classified as ‘possible or probable’ ADR associated with SP and/or AS. Seventeen cases were excluded in the final household cost analysis due to failure to provide complete treatment costing information; ten were unable to recall any of the expenses incurred and the remaining seven had incomplete cost information. Since both cases classified as ‘probable’ or ‘possible’ ADR events had incurred some costs during treatment, they were all included in the cost analysis. Table 4 summarizes the characteristics of these ADR patients and their treatment-seeking behaviour. Of all 50 patients with complete cost information, 37 patients incurred some expenses. Twenty-three patients financed their costs through out of pocket expenditures, with only three cases paying through community-based or government-employee insurance schemes. Five ADR patients reported having made informal payments at the place they sought care; mainly at government health facilities.

**Table 4:** Patient characteristics, care-seeking and type of payments: (Includes all probable and possible cases with complete cost information)

	**Rufiji District**	**Kilombero/Ulanga Districts**	**Total**
**Number of cases with complete cost information**	**28**	**22**	**50**
***Health outcomes on day of interview:***			
Fully recovered	21	17	**38**
Partially-recovered	3	2	**5**
Patients died	4	3	**7**
***Patient characteristics:***			
Female	17	13	**30**
Age under-five years	6	6	**12**
***Patient care-seeking sources:***			
Public health facilities	19	12	**31**
Faith based/NGO facilities	6	8	**14**
Private drug shop outlets	2	1	**3**
Obtained drugs from family & neighbours	1	1	**2**
***Treatment expenditure pattern***			
Patients with no expenses	11	2	**13**
Patients with one or multiple expenses	17	20	**37**
***Payment mechanisms for patients with treatment expenses N = 37***			
Only cash that was to hand/past savings	10	13	**23**
Health insurance (community/employer)	1	2	**3**
Cash to hand and/or selling household assets or part of farm harvest	1	3	**4**
Borrowed from friends or relatives	5	2	**7**
***Report of any informal payments***			
Made informal payments at the place of care	2	3	**5**

Forty-five patients reported visiting government or private health facilities, three obtained drugs from private drug outlets only and two obtained drugs from relatives and friends. Of those visiting healthcare providers, patients made one to four visits to single or different providers. A total of 77 visits to different healthcare providers were made. The majority (44 (57%)) were to public health facilities, mainly government dispensaries and health centres, followed by 24 (31%) to faith-based and NGO facilities, with only seven (9%) to commercial retail drug stores. There were two (3%) patients who obtained drugs from their relatives and friends only. Furthermore, two patients made four visits each, three patients made three visits, 15 made two visits, and the remaining 30 patients sought care from one provider. Reasons given for multiple visits included poor case diagnosis, slow recovery and lack of appropriate drugs or drug stock-outs. For the two patients making four visits, one patient made two visits to a government dispensary, one visit to a mission dispensary, one visit to a disability hospital in Dar es Salaam and a government district hospital in Rufiji District. The other visited a drug shop, a government dispensary, a government health centre and finally a mission hospital.

Of all 50 ADR patients with complete cost information, 22 (44%) were admitted at some point during the illness episode, with admissions lasting from 24 hours up to 60 days. The mean number of admission days was 14.5 (SD-17.12) with a median of 7.5 days and an interquartile range (IQR) of 13 days.

### Household costs of treatment-seeking

Total household costs per episode are shown in Table 5 including those with and without expenditure, and those having fully recovered, partially recovered or died. Mean expenditure per episode was US$24.15 (SD 40.00) with a median of US$10.00 and a range from US$0 to 226.04. If only those who reported some expenditure are included, the mean outlay per episode was US$31.78 (SD 43.18), with a range of US$0.22 to 226.04 and a median of US$19.59. Considering all cases, there was some variation in expenditure by patient characteristics (though results should be interpreted with caution given the small sample size). Median expenditure was significantly higher for ‘probable’ ADR events compared to ‘possible’ ADR (p = 0.079), for admitted cases compared to those managed as outpatients (p = 0.000), and for cases which had taken SP alone compared to SP + artesunate (p = 0.013), but there were not corresponding significant changes in mean expenditure. Mean and median costs were significantly higher for patients who made multiple provider visits (T-test for the means and Mann–Whitney median test of p < 0.001 and p = 0.016, respectively for those making single visit compared to four visits). Finally, the mean expenditure per visit was US$15.68 (SD 20.56) with a median of US$8.18.

**Table 5:** Household treatment expenditure per ADR episode (2005 USD)

**Expenses for patients with and without expenditures**				
	**Number of episodes**	Mean^*^	**Median**^**#**^	**Range**
Episodes with complete cost information (with and without expenses)	50	24.15	10	0.00 – 226.04
***ADR Classification:***				
Probable	31	28.85	14.61~	0.00 –226.04
Possible	19	16.48	3.48~	0.00 – 115.91
***Treatment costs by admission status:***				
Admitted	22	47.18**	30.83**	0.00 – 226.04
Not admitted	28	6.05**	2.61**	0.00 – 35.39
***Treatment costs by survivorship***				
Costs for patients surviving	43	17.15	7.80^	0.00 – 125.87
Costs for Patients dying	7	63.89	28.74^	0.00 – 226.04
***Number of visits:***				
One	30	7.24**	2.23^	0.00 – 44.04
Two	15	32.01**	28.74^	1.74 – 115.91
Three	3	72.09	46.48	43.91 – 125.87
Four	2	146.93**	146.93^	67.83 – 226.04
***Study district:***				
Rufiji	28	21.68	3.61^	0.00 – 226.04
Kilombero / Ulanga	22	27.29	21.80^	0.00 – 115.91
***Age group:***				
Under 5 years	13	11.60	5.99	0 – 32.91
5 years or over	37	28.11	10.78	0 – 226.04
***By drug use***				
AS only	1	9.74	9.74^	9.74 – 9.74
SP only	35	29.02	19.61^	0 – 226.04
SP + Artesunate	14	13.00	3.61^	0 – 125.87

Note: *Students t-test between groups &^**#**^Wilcoxon rank sum (Mann- Whitney) test.

**Significant between one and four visits, and between two and four visits at 1% level.

^Significant between groups and/or between one and four visits and between two and four visits at 5% level.

~Significant between groups and/or between one and four visits, and between two and four visits at 10% level.

Moreover, ADR treatment expenditure by type of health care providers visited was explored. Overall, faith-based and NGO facilities were the most expensive with median expenditure of US$22.41 per visit, followed by private drug retailers at US$15.40, and government health facilities (median of US$5.56) (Table 6). There was a significant difference between both mean and median expenditures at government health facilities and faith-based/NGO facilities (T-test for the means: p = 0.0160 and Mann–Whitney median test: p = 0.0002).

**Table 6:** **Household treatment expenditure per visit by provider type (2005 USD)**^**1**^

**Source of care visited**	**Number of visits**	**Mean**	**Median**	**Range**
Government facilities	44	11.79*	5.56**^**	0 – 87.74
Faith based and NGO facilities	24	24.71*	22.41^	0.09 – 81.04
Private drug outlets	7	13.68	15.4	0.56 – 33.83
Drugs from relatives and friends	2	0	0	0.00 – 0.00
**All patients visits**^**1**^	**77**	**15.68**	**8.18**	**0 – 87.74**

^1^ Includes those with and without any expenditure.

* Student t-test significant at 1% level between Government and Faith based /NGO Health Facilities.

**^** Mann–Whitney test significant between the two groups at 5% level: Government vs. Faith based / NGO Health Facilities.

Drug costs constituted the largest component of total treatment expenditures at 43.3%, followed by transport, food and accommodation (32.9%), and registration, consultation, laboratory tests and other fees (23.8%) (data not shown). In one case the family of a deceased patient reported hiring a vehicle to transport the patient from Dar es Salaam to Rufiji District costing them about US$130. The expenditure pattern was fairly similar for both government and non-governmental facilities, with expenditure at private drug retailers being mostly on drugs alone. For those reporting informal expenses, payments ranged from US$0.21 to 4.34 per visit, with a mean informal payment of US$1.07 and median of US$0.36.

For the four patients reporting to have sold some of their assets or farm harvests, they mentioned selling various items including animals, bags of rice and corn and household furniture. Additionally, seven had to borrow from relatives or friends to pay their treatment bills.

## Discussion

ADR occurrence rates in rural Tanzania were estimated at 11.6 and 25.0 due to exposure to SP in Rufiji and Kilombero/Ulanga respectively. Incidence due to use of AS/SP combination was substantially lower at 5.6 per 100,000 exposures. A number of reasons might have been responsible for this trend, including differences in quality of care, frequency of drug use between the sites and a relatively sporadic supply chain for the AS/SP combination drugs. At least in Kilombero and Ulanga district, there was a project engaged in improving access to anti-malarials which might have also influenced the use of sulphonamide containing anti-malarials amongst the targeted population [45]. Our study design may also have been somehow biased as it was more geared to identify ADR cases caused by use of SP monotherapy than AS/SP combination drugs. Following the national anti-malarial policy change, there were anecdotal reports of ADR events caused by SP drugs resulting to general public outcry and resistance in using SP drugs for malaria treatment. Therefore, ADR SOs in districts with SP monotherapy enjoyed closer supervision and follow-up of their activities than those in Rufiji which includes the highly inaccessible Rufiji delta area where reporting and tracing cases for confirmation might have been more difficult. Seven of the 67 cases successfully traced and clinically classified died for reasons associated with ADR. Of those dying, four were recorded in Rufiji and three were from Kilombero and Ulanga districts. Estimations of ADR occurrence rates were based on cases classified as ‘probable’ ADR events only, and would have been higher if ‘possible’ ADR had also been included. It is also important to note that most of the ADR cases reported were associated with SP and SP/AS (but none for AS alone).

These findings may be compared with studies conducted in other malaria-endemic settings including Malaysia, Peru and Malawi [16,17,30]. In the Malaysian study, ADR incidence was estimated at 2.4 per 100,000 exposures to SP. SP was used prophylactically by almost a half of those exposed and almost certainly involved repeated dosing [17]. The study in Peru followed up patients exposed to the combination of SP plus artesunate in selected clinics for two years, with 8% reporting some associated ADR, although very mild reactions were included in the ADR definition [16]. A study in Blantyre, Malawi beginning early 2001 used a definition more comparable to that used in this study, and estimated ADR associated with use of SP or trimethoprim-sulphamethoxazole (cotrimoxazole) at 0.3 to 8.4 per 100,000 exposures [30]. ADR incidence among HIV positive persons was higher at 20.4 per 100,000 exposures. This study did not collect information on HIV status during the IMPACT-Tz study, however, other sources indicate that HIV prevalence rates at the time ranged from 4 to 7% across the study districts [46]. The studies from Peru and Malawi relied on health facility-based surveillance only, with no active community surveillance. The authors of the Malawi study in particular acknowledged this limitation and suggested that that the number of ADR cases might have been under-reported, especially in areas where access to health facilities was difficult [30]. Following the nature of active surveillance component of this study, it is not surprising that incidence rates are higher. Studies on healthcare-seeking behaviour in the three study districts have reported that government health facilities see less than 60% of all healthcare seekers with fever/malaria, with many patients purchasing drugs at drug shops, general shops and small kiosks [40,47], an indication that reliance on passive surveillance only is likely to miss some cases. However, it is possible that the figures presented in this study are underestimated, as recall bias and the assumption of homogeneity in the level of exposure to these drugs may have led to under-reporting during active surveillance, especially of more minor ADR. It is also important to note that this study’s surveillance estimates were based on areas with routine demographic surveillance where a range of health interventions are continually evaluated. It is possible that exposure to anti-malarial drugs in the study sites was therefore higher than might be reported from the rest of the country.

Analysis of the household costs of treating probable and possible ADR events resulting from use of anti-malarials showed that despite these events being rare occurrences, their associated treatment expenses can have important economic consequences. Mean treatment expenditure for an ADR episode was US$24.45 with a median of US$10.00, with average expenditure rising rapidly with number of providers visited. Average household costs of care were ten-fold higher than those reported for treating an episode of fever or malaria [48,49]. For example, studies conducted in the same districts in mid-2001 reported a mean household expenditure of US$0.30 and a median of US$0.06 (all in 2001 prices) per fever episodes [50].

Furthermore, some patients with ADR endured lengthy periods of illness as indicated by median admission days of 7.5 with the longest hospital admission lasting 60 days. Data were not collected on the total number of disability days caused by ADR events, but it seems likely that the number of productive days lost was higher than those usually reported in most malaria studies which do not consider ADR [48,49]. By the time this study was completed, of the 50 patients with complete cost information, five (10%) patients said they were yet to fully recover.

The costs for treating ADR resulting from use of SP monotherapy were found to be higher than those from ACT use. However, this may be confounded by the different health financing policies in place in the two study areas during the study period. In Kilombero and Ulanga Districts where SP monotherapy was routinely delivered as first-line treatment, government facilities had started implementing user fees for all patients except pregnant mothers and children aged below five years in early 2003. In contrast, in Rufiji District, where SP/AS was available, services at government-owned facilities were officially free until late 2005 when user fees were introduced for the first time.

There are indications that the costs associated with treatment of drug reactions placed substantial financial burden on the households concerned. Of the 37 ADR patients who paid money while seeking treatment, only six patients made payments through a health insurance scheme, an indication of very low health insurance coverage in these areas. Additionally, eight patients reported having to sell part of their farm harvests or household assets; and two had to borrow money to finance their treatment. An alternative way to understand the magnitude of the treatment burden placed on households because of ADR events is by comparison with total household per capita income or expenditure. Tanzania’s GDP per capita was estimated at US$326 in 2005 [51], and median monthly household expenditure in the HDSS study sites ranged from US$77 to 96 with 75% of expenditure on food [34]. Following the WHO criteria for catastrophic expenditures, households spending 50% or more of their non-food expenditure on healthcare are likely to be impoverished by the cost [52,53]. Other studies have defined household expenditures as catastrophic whenever household healthcare spending exceeds 40% of income remaining after subsistence needs are met [54,55]. In either case, these findings indicate that a majority of treatment expenditures on adverse events could be considered catastrophic.

Finally, it is important to note that full cost information was only available for 50 patients classified as ‘possible or probable’ ADR events. This is an inherent problem with studying rare events. Some respondents had to recall costs over a relatively long period compared to standard household surveys, which may have increased the likelihood of recall bias. However, perhaps reflecting the fact that ADR are unusual and possibly catastrophic events, during data collection interviewees did not have difficulties in remembering treatment sought and costs incurred. Meanwhile it is also important to note that five cases included in the costing analysis had not fully recovered on the day of interview. This may have slightly underestimated the total cost of their treatment. However, all except one had already obtained all their drugs for treating their conditions and were unlikely to visit healthcare facilities or drug shops for additional treatment to the same illness.

While these drugs were found to be generally safe, this study underscores the importance of improving ADR surveillance for anti-malarial drugs. The use of active surveillance especially in places with HDSS research platforms may play an important part in enhancing post marketing drug safety surveillance. Efforts to increase access to effective anti-malarials should go hand in hand with implementation of effective drug safety surveillance across nations [28], especially given the new generation of anti-malarial drugs in the pipeline and concerns about fake and substandard anti-malarials [26,56].

## Conclusions

Household ADR occurrence rates in rural Tanzania were estimated at 11.6 and 25.0 per 100,000 exposures to SP in Rufiji and Kilombero/Ulanga Districts respectively, and at 5.6 per 100,000 exposures to AS/SP in Rufiji. Both passive and active surveillance methods proved to be feasible for carrying out SP and/or AS related ADR surveillance. Active surveillance provided an important complement to the health facility-based passive surveillance, given the widespread practice of self-medication in Tanzania. The costs of treating patients suffering from ADR were high and potentially catastrophic to the majority of poor households, with particularly high costs for those reporting hospital admissions. Efforts should be made to both strengthen pharmacovigilance monitoring across Africa and to identify strategies to reduce the economic burden for households suffering from ADR.

## Competing interests

All authors of this article declare that they have no competing interests.

## Authors’ contributions

JDN, AMK, CCG, JAM, BKK, JG PBB SMA and SPK conceived and designed the study. JDN, AMK, CCG, JAM, EK, SMA & SPK executed the study. JDN, CCG, JAM, AMK & SPK analysed the data and JDN wrote the first draft of the report. All authors contributed to the first and second draft of the report. All authors approved the final report in its current format.
